# CXCL1 can be regulated by IL-6 and promotes granulocyte adhesion to brain capillaries during bacterial toxin exposure and encephalomyelitis

**DOI:** 10.1186/1742-2094-9-18

**Published:** 2012-01-23

**Authors:** Monica Roy, Jean-François Richard, Aline Dumas, Luc Vallières

**Affiliations:** 1 Department of Endocrinology and Genomics, Laval University Hospital Research Center, 2705 Laurier Boulevard, Quebec G1V 4G2, QC, Canada

**Keywords:** Neuroinflammation, Neuroimmunity, Chemotaxis, Myeloid cells, Polymorphonuclear cells, Neutrophils, Cerebral endothelium, Autoimmunity.

## Abstract

**Background:**

Granulocytes generally exert protective roles in the central nervous system (CNS), but recent studies suggest that they can be detrimental in experimental autoimmune encephalomyelitis (EAE), the most common model of multiple sclerosis. While the cytokines and adhesion molecules involved in granulocyte adhesion to the brain vasculature have started to be elucidated, the required chemokines remain undetermined.

**Methods:**

CXCR2 ligand expression was examined in the CNS of mice suffering from EAE or exposed to bacterial toxins by quantitative RT-PCR and *in situ *hybridization. CXCL1 expression was analyzed in IL-6-treated endothelial cell cultures by quantitative RT-PCR and ELISA. Granulocytes were counted in the brain vasculature after treatment with a neutralizing anti-CXCL1 antibody using stereological techniques.

**Results:**

CXCL1 was the most highly expressed ligand of the granulocyte receptor CXCR2 in the CNS of mice subjected to EAE or infused with lipopolysaccharide (LPS) or pertussis toxin (PTX), the latter being commonly used to induce EAE. IL-6 upregulated CXCL1 expression in brain endothelial cells by acting transcriptionally and mediated the stimulatory effect of PTX on CXCL1 expression. The anti-CXCL1 antibody reduced granulocyte adhesion to brain capillaries in the three conditions under study. Importantly, it attenuated EAE severity when given daily for a week during the effector phase of the disease.

**Conclusions:**

This study identifies CXCL1 not only as a key regulator of granulocyte recruitment into the CNS, but also as a new potential target for the treatment of neuroinflammatory diseases such as multiple sclerosis.

## Background

Myelin-reactive CD4^+ ^T lymphocytes play a critical role in the pathogenesis of multiple sclerosis and its animal model, EAE [1]. A basic principle in immunology states that these cells do not act alone, but rather in concert with different populations of myeloid phagocytes, which activate them by presenting antigens and producing proinflammatory molecules, and which execute effector functions. The phagocytes best known to be involved in EAE are monocyte-derived CD11c^+ ^dendritic cells [2-5] and Ly6C^+ ^macrophages originating from either microglia or monocytes [2,4,6,7]. In addition, mounting evidence suggests the involvement of a third population of phagocytes, namely granulocytes. Indeed, it has been reported that granulocytes massively infiltrate the CNS of EAE mice [8-17], and that EAE is markedly attenuated in mice either treated with antibodies against the granulocyte proteins CXCR2 and Ly6G, treated with a small molecule antagonist of CXCR2, or genetically manipulated to suppress CXCR2 [12,18-20]. Therefore, granulocytes and the extracellular signaling pathways that control them represent novel potential therapeutic targets for multiple sclerosis.

We have uncovered a population of rod-shaped granulocytes that patrol the CNS vasculature by crawling on the luminal endothelial surface [17,21,22]. These cells are recruited in greater number in mice suffering from EAE or exposed to bacterial products such as LPS and PTX [17,21]. According to the classical model, the mechanism underlying this recruitment includes the following steps: 1) proinflammatory cytokines induce the expression of chemokines and adhesion molecules on the endothelial surface; 2) these chemokines activate granulocytes by promoting the conversion of integrins from a low-affinity to a high-affinity state; and 3) the latter integrins allow the firm attachment of granulocytes to endothelial adhesion molecules [23]. So far, we have gathered evidence that PTX induces ICAM1 expression in brain capillaries indirectly through IL-6, and that granulocytes bind to these vessels through interaction between Mac1 (integrin αMβ2) and intercellular adhesion molecule 1 (ICAM1) [17]. In contrast, granulocyte adhesion in response to LPS is independent of IL-6, but dependent on TNF and IL-1β [17,21]. An important question that remains to be addressed is what chemokine(s) control granulocyte adhesion in the cerebral microvasculature.

The G-protein-coupled receptor CXCR2 is crucial for granulocyte infiltration into the brain parenchyma, as demonstrated using CXCR2-knockout mice exposed to LPS or *Staphylococcus aureus *[24,25]. However, it is still unclear whether CXCR2 intervenes during adhesion and/or transmigration. CXCR2 binds to chemokines of the CXC family containing the glutamate-leucine-arginine (ELR) motif (i.e., CXCL1, CXCL2, CXCL3, CXCL5, and CXCL7 in mouse and human, in addition to CXCL6 and CXCL8 in human only) [26]. This family also comprises mouse CXCL15, which does not appear to bind CXCR2 [27]. Both CXCL1 and CXCL2 are upregulated in the CNS of mice suffering from endotoxemia [24] or EAE [13,14,16,28-30]. Such information is not available for the other CXCR2 ligands, leading to question the relative importance of these molecules in granulocyte recruitment into the CNS.

The primary objective of the present study was to identify the main CXCR2 ligand responsible for granulocyte adhesion to brain capillaries during EAE and after exposure to bacterial toxins, especially PTX, which is commonly used as an adjuvant to induce EAE [31], but whose mechanism of action is still not fully understood. The second objective was to examine whether the effect of PTX on CXCL1 expression is mediated by IL-6, a cytokine essential for EAE development [32-36] and previously identified as mediating the effect of PTX on endothelial ICAM1 expression [17].

## Methods

### Mice

Experiments were performed on male mice aged 8-10 weeks with the approval of our institutional animal ethics committee. The animals were obtained from Charles River Laboratories (C57BL/6 mice) or The Jackson Laboratory (IL-6-knockout and wild-type mice with a C57BL/6 background). They were housed individually in ventilated cages and acclimated for at least 1 week before use.

### Toxin injection

Mice were injected intraperitoneally with 20 μg/kg PTX (List Biological Laboratories) or 1 mg/kg LPS from *Escherichia coli *O55:B5 (Sigma-Aldrich) both diluted in PBS. Control mice were injected with PBS only.

### EAE induction and clinical evaluation

Mice were injected subcutaneously on day 0 with 200 μl (100 μl/site) of emulsion containing 300 μg of myelin oligodendrocyte glycoprotein peptide 35-55 (AnaSpec) dissolved in saline and mixed with an equal volume of complete Freund's adjuvant containing 500 μg of killed *Mycobacterium tuberculosis *H37 RA (Difco Laboratories). The animals were also injected intraperitoneally with PTX (20 μg/kg) immediately and 48 h after the first immunization. Clinical signs were monitored daily and scored as follows: 0, no detectable sign; 0.5, partially limp tail; 1, paralyzed tail; 2, loss in movement coordination and hind limb paresis; 2.5, one hind limb paralyzed; 3, both hind limbs paralyzed; 3.5, hind limbs paralyzed and weakness in forelimbs; 4, fore limbs paralyzed; 5, moribund or dead [31].

### Treatment with neutralizing antibodies

Mice were injected via a tail vein with the following antibodies (all purchased from R&D Systems) at a concentration of 4 or 20 mg/kg in PBS: anti-CXCL1 (rat IgG_2A_), anti-CXCL2 (rat IgG_2B_), anti-CXCR2 (rat IgG_2A_), and isotype control antibodies (rat IgG_2A_). A single injection of these antibodies was given 1 h before toxin injection, whereas daily injections were given from day 7 to day 13 after EAE induction.

### RNA isolation

Total RNA was isolated from tissues and cultured cells by homogenization in TRI-reagent (Sigma-Aldrich) followed by purification using the GenElute Mammalian Total RNA Miniprep Kit (Sigma-Aldrich). RNA integrity and quantity were assessed using the Bioanalyzer 2100 capillary electrophoresis system (Agilent Technologies) and the NanoDrop 2000 spectrophotometer (Thermo Scientific), respectively.

### Quantitative RT-PCR

First strand cDNA was generated from 2 μg of total RNA using Superscript III (Invitrogen) with random hexamer and 20-mer oligo-dT primers, then purified with the GenElute PCR Clean-Up Kit (Sigma-Aldrich). The product (20 ng) was analyzed using the LightCycler 480 system with the SYBR Green I Master mix according to the manufacturer's instructions (Applied Biosystems). The primers were as follows: CXCL1, 5'-ATCCAGAGCTTGAAGGTGTTG-3' and 5'-GTCTGTCTTCTTTCTCCGTTACTT-3'; CXCL2, 5'- ATGCCTGAAGACCCTGCCAAG-3' and 5'-GGTCAGTTAGCCTTGCCTTTG-3'; CXCL3, 5'-CATCCAGAGCTTGACGGTGAC-3' and 5'-CTTGCCGCTCTTCAGTATCTTCTT-3'; CXCL5, 5'-ACAGTGCCCTACGGTGGAAGT-3' and 5'-CGAGTGCATTCCGCTTAGCTT-3'; CXCL7, 5'-GGAAAATCTGATGGCATGGAC-3' and 5'-CAGGCACGTTTTTTGTCCATTCT-3'; SOCS3, 5'-GAGAAGATTCCGCTGGTACTG-3' and 5'-GCAGCTGGGTCACTTTCTCATA-3'; ICAM1, 5'- CCCAAGGAGATCACATTCACG-3' and 5'-TTCCAGGGAGCAAAACAACTTCT-3'. The PCR conditions consisted of 45 cycles of 10 sec at 95°C (denaturation), 10 sec at 60°C (annealing), 12 sec at 72°C (elongation), and 5 sec at 78°C (reading). The number of cDNA copies was determined using the second derivative method as described previously [37].

### Immunostaining

Immunohistochemistry was performed as described previously [21] using the following primary antibodies: rat anti-CD3 (1:500, Serotec), rat anti-CD31 (1:500, BD Biosciences), rat anti-CD45 (1:1000, BD Biosciences), rabbit anti-Iba1 (1:2000, Wako Chemicals), rabbit anti-GFAP (1:1000, Millipore), and rat anti-Ly6G (1:5000, BD Biosciences).

### *In situ *hybridization

Brain sections were analyzed by *in situ *hybridization as described previously [38]. Combined *in situ *hybridization and immunohistochemistry was performed according to a previously described protocol [39].

### Stereology

Cells were counted using the optical fractionator methods as described previously [21].

### Flow cytometry

Blood samples were blocked for 15 min with 5 μg/ml anti-CD16/CD32 antibody (BD Biosciences) and stained for 30 min on ice with the following antibodies (1 μl each per 10^6 ^cells): rat anti-7/4-RPE (Serotec), rat anti-CD45-FITC (BD Pharmingen), rat anti-CD115-APC (eBioscience), and rat anti-Ly6G-PerCP-Cy5.5 (BD Pharmingen). After hemolysis and fixation with the Whole Blood Lysing Reagent Kit (Beckman Coulter), cells were analyzed with a BD FACSCanto II flow cytometer and FlowJo software (Tree Star).

### Cell culture

bEnd.3 cerebral endothelial cells (American Type Culture Collection) were cultured in six-well plates in DMEM containing 10% FBS, 100 U/ml penicillin, and 100 μg/ml streptomycin (Sigma-Aldrich). At confluence, the medium was replaced with fresh medium supplemented or not with mouse IL-6 (10 ng/ml, R&D Systems), mouse IL-6 receptor α (IL-6Rα; 1 μg/ml, R&D Systems), and/or rat anti-mouse-IL-6 antibody (40 μg/ml, R&D Systems). The cells were lysed 3 h later for RNA isolation. Supernatant was collected for quantification of CXCL1 using an ELISA kit (R&D Systems).

### Statistical analyses

Data are expressed as mean ± standard error. Means were compared using the unpaired Student's *t *test, one-way ANOVA, or two-way ANOVA. Alternatively, the Kruskal-Wallis test was used when the distribution was abnormal. Student's *t *tests or Wilcoxon tests were performed for *post hoc *multiple comparisons. All these analyses were performed using JMP (SAS Institute) with a significance level of 5%.

## Results

### CXCL1 is the main CXCR2 ligand expressed in the inflamed CNS

**Figure 1:**
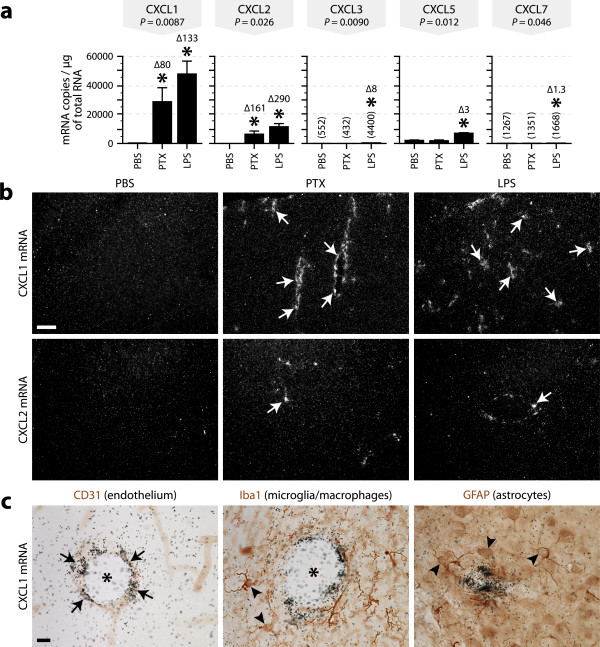
**Upregulation of CXCR2 ligands in the brain after exposure to bacterial toxins**. ***a***, Quantification of the mRNAs encoding each of the CXCR2 ligands by quantitative RT-PCR using RNA samples prepared from the brains of mice killed 6 h after intraperitoneal injection of PBS, PTX (20 μg/kg), or LPS (1 mg/kg). The means were compared with the Kruskal-Wallis test (*P*-values as indicated) followed by *post hoc *Wilcoxon tests. For the weakly expressed genes, the means are indicated in parentheses. *Significantly different from the PBS group. Δ = fold change relative to PBS. *n *= 5 per group. ***b***, Dark-field micrographs showing *in situ *hybridization signals for CXCL1 or CXCL2 mRNA in the cerebral cortex of mice treated with PTX or LPS. No signal was detected in control mice (PBS). Arrows show clusters of emulsion grains indicating the presence of cells expressing the transcripts. Scale bar = 100 μm. ***c***, Double labeling for CXCL1 mRNA (black grains, *in situ *hybridization) and different cell type-specific markers (red-brown, immunohistochemistry) in brain sections from PTX-treated mice. Arrows indicate double-labeled cells. Arrowheads show examples of immunostained cell bodies negative for CXCL1 mRNA. *Blood vessel lumen. Scale bar = 10 μm.

To determine which CXCR2 ligands are expressed in the brain during inflammatory conditions, we first measured by quantitative RT-PCR the mRNA levels of these ligands in the brains of mice killed 6 h after intraperitoneal injection of PTX (20 μg/kg), LPS (1 mg/kg), or control solution (PBS). As shown in Figure 1a, both toxins upregulated CXCL1 and CXCL2 mRNAs by ≥ 80 times compared to the basal levels. The transcripts of CXCL1 were ~4 times more abundant than those of CXCL2. In contrast, only LPS upregulated CXCL3, CXCL5, and CXCL7 mRNAs, albeit by no more than 8 times. These transcripts were respectively 108, 7, and 287 times less abundant than those of CXCL1 in LPS-treated mice.

To confirm the above results and examine the distribution of the cells expressing CXCR2 ligands, we next analyzed brain sections by *in situ *hybridization. Many cells positively labeled for CXCL1 mRNA were detected throughout the brains of mice exposed to either toxin (Figure 1b), whereas only a few positive cells were detected for CXCL2 mRNA (Figure 1b) and none for CXCL3, CXCL5, or CXCL7 mRNA (data not shown). In general, the labeled cells appeared to be distributed along blood vessels, especially those of large caliber. To confirm this observation, we double labeled brain sections for CXCL1 mRNA and cell type-specific markers. The hybridization signals colocalized with the endothelial marker CD31, but not with the microglial marker Iba1 or the astrocytic marker GFAP (Figure 1c). Similar observations were made for CXCL2 mRNA (data not shown).

**Figure 2:**
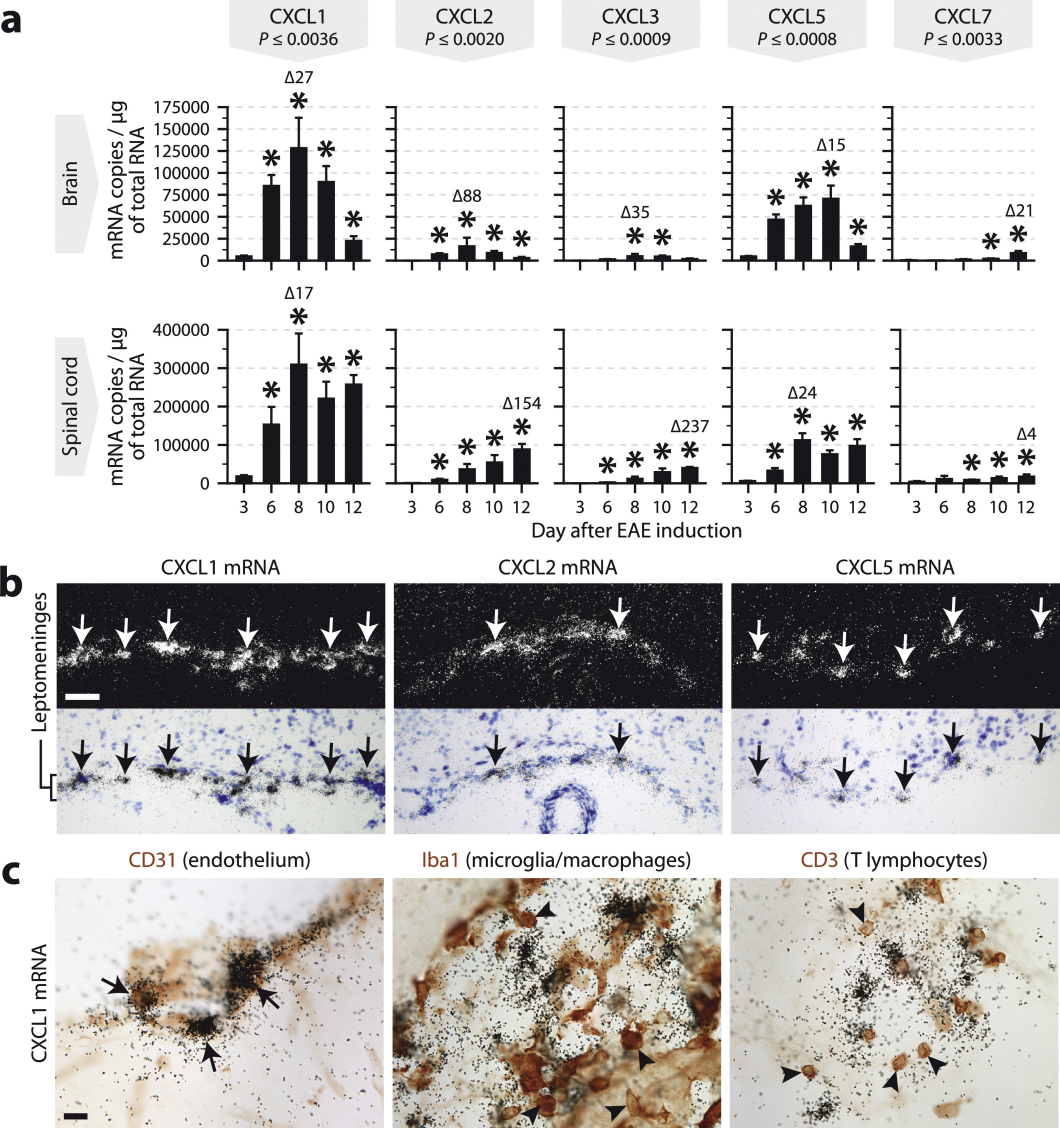
**Upregulation of CXCR2 ligands in the brain and spinal cord during EAE**. ***a***, Quantification of the mRNAs encoding each of the CXCR2 ligands by quantitative RT-PCR using RNA samples prepared from the brains or spinal cords of mice killed at the indicated time intervals after EAE induction (as shown in Additional file 1: Figure S1, the first clinical symptoms were observed on day 8). The means were compared with the Kruskal-Wallis test (*P*-values as indicated) followed by *post hoc *Wilcoxon tests. *Significantly different from day 3. Δ = fold change relative to day 3. *n *= 6 (days 3, 6, and 10), 5 (day 8), or 3 (day 12). ***b***, Dark-field and bright-field micrographs showing *in situ *hybridization signals for CXCL1, CXCL2, or CXCL5 mRNA in the leptomeninges of EAE mice killed 6 days after EAE induction. Arrows show clusters of emulsion grains indicating the presence of cells expressing the transcripts. Scale bar = 50 μm. ***c***, Double labeling for CXCL1 mRNA (black grains, *in situ *hybridization) and different cell type-specific markers (red-brown, immunohistochemistry) in brain sections from EAE mice. These images were taken at the level of the leptomeninges. Arrows indicate double-labeled cells. Arrowheads show examples of immunostained cell bodies negative for CXCL1 mRNA. Scale bar = 10 μm.

To examine whether a comparable upregulation of CXCR2 ligands occurs in EAE, we repeated the experiments described above using RNA and tissue samples obtained from mice killed from 3 to 12 days after EAE induction. As observed in LPS-treated mice, all of the CXCR2 ligands were transcriptionally upregulated in the brains and spinal cords of EAE mice, the most highly expressed being CXCL1 (Figure 2a). With a marked increase noted from day 6, their expression at day 12 was still maximal in the spinal cord, but tended to decrease in the brain. In contrast to what we observed with the toxins, the signals detected by *in situ *hybridization were mainly distributed in the choroid plexus and leptomeninges (Figure 2b), where they colocalized with CD31 staining and where numerous infiltrating T cells were present (Figure 2c). Overall, the results presented thus far indicate that CXCL1 is the main CXCR2 ligand expressed in the CNS in response to different inflammatory stimuli and originates from the vasculature.

### IL-6 induces CXCL1 transcription in endothelial cells

**Figure 3:**
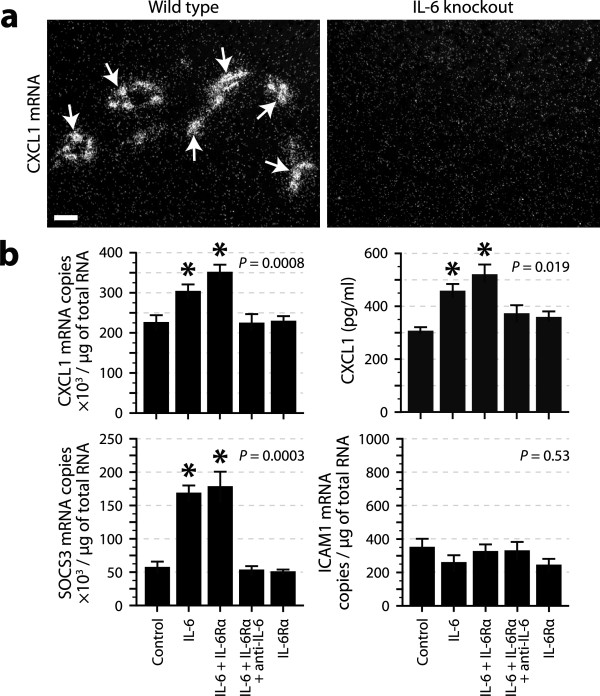
**Regulation of CXCL1 expression by IL-6 in cerebral endothelial cells *in vivo *and *in vitro***. ***a***, Dark-field micrographs of brain sections collected from IL-6 knockout and wild-type mice killed 6 h after intraperitoneal injection of PTX (20 μg/kg) and analyzed for CXCL1 mRNA by *in situ *hybridization. Positively labeled cells (arrows) are seen only in the wild-type mouse (representative of 6 mice per genotype). Scale bar = 50 μm. ***b***, Quantification of CXCL1 expression by quantitative RT-PCR or ELISA in cultures of bEnd.3 cerebral endothelial cells exposed for 3 h to IL-6 (10 ng/ml), soluble IL-6 receptor (IL-6Rα, 1 μg/ml), and/or neutralizing anti-IL-6 antibody (40 μg/ml). SOCS3 and ICAM1 mRNAs were used as a positive or negative control, respectively. The PCR data were compared with the Kruskal-Wallis test (*P*-values as indicated) followed by *post hoc *Wilcoxon tests. The ELISA data were compared using ANOVA (*P*-value as indicated) followed by *post hoc *Student's *t *tests. *Significantly different from the control group. *n *= 6 per condition.

We have previously shown that PTX increases ICAM1 expression in the cerebral endothelium indirectly through IL-6, which acts post-transcriptionally, but not transcriptionally [17]. To test whether IL-6 also regulates CXCL1 expression, we compared the levels of CXCL1 mRNA in the brains of IL-6-knockout and wild-type mice injected with PTX by *in situ *hybridization. Many strong hybridization signals were observed in the wild types, but no signal was detected in the knockouts (Figure 3a). To complement this experiment, we cultured cerebral endothelial cells in the presence of IL-6 for 3 h, and found an increased expression of CXCL1 both at the mRNA and protein levels (Figure 3b). Although this effect was not enhanced by the addition of soluble IL-6 receptor, it was totally inhibited by pre-incubation with an anti-IL-6 antibody, confirming the specificity of the results. The validity of this experiment is also supported by the observations that SOCS3 and ICAM1 mRNAs were respectively upregulated and unaffected by IL-6, as expected from previous studies [17,40]. Altogether, these findings demonstrate that IL-6 can regulate CXCL1 expression by acting at the transcriptional level.

### CXCL1 promotes granulocyte adhesion to cerebral blood vessels

**Figure 4:**
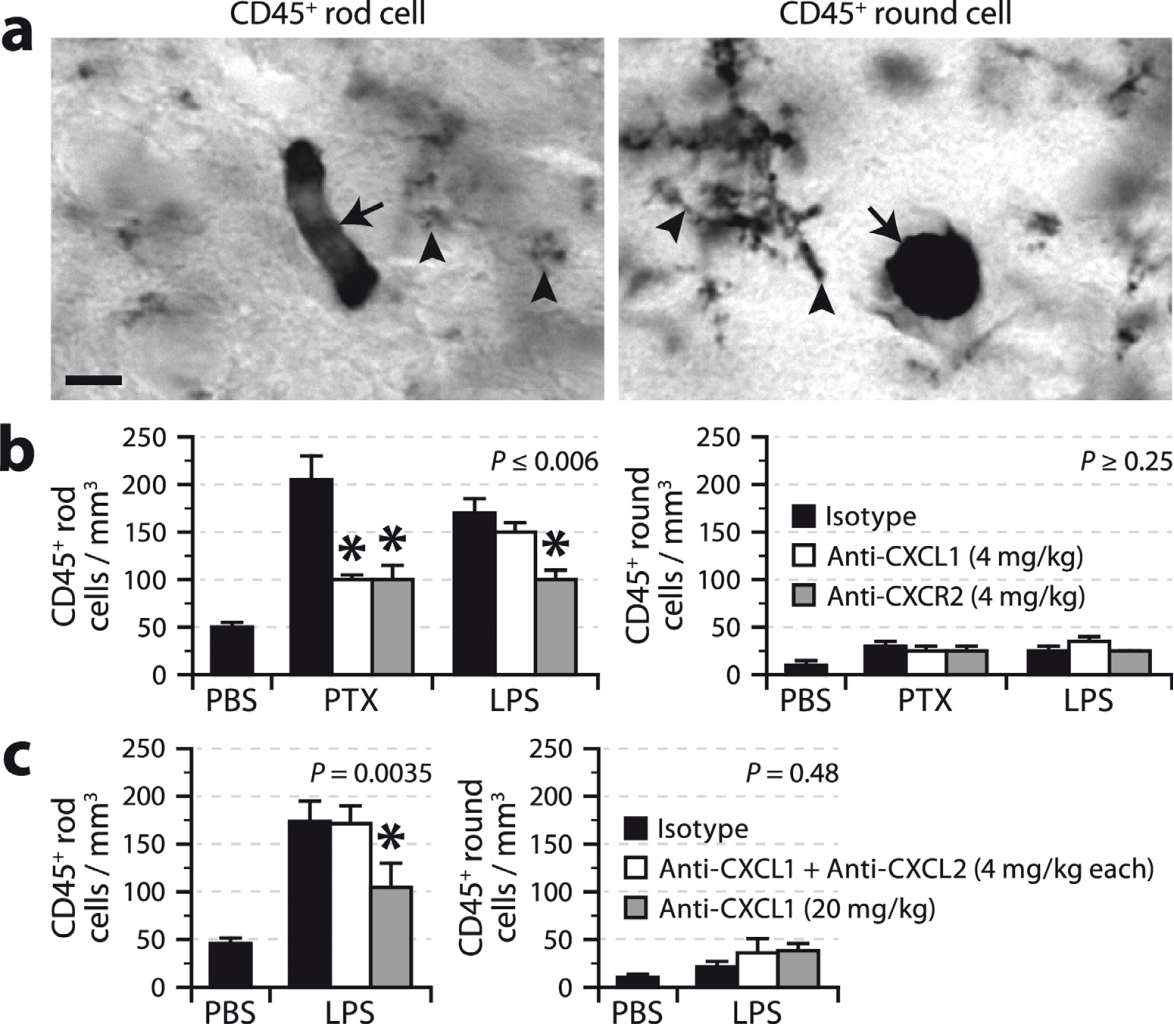
**Contribution of the CXCL1-CXCR2 axis to leukocyte adhesion within the cerebral vasculature in response to bacterial toxins**. ***a***, Micrographs of intravascular leukocytes (arrows) with a rod-shaped or rounded morphology in mouse brain sections stained for CD45 by immunohistochemistry. Arrowheads = microglial processes. Scale bar = 5 μm. ***b***, Counts of CD45^+ ^intravascular leukocytes (rod-shaped and round) in the cerebral cortex of mice treated with anti-CXCL1, anti-CXCR2, or isotype antibody (4 mg/kg), and killed 6 h after intraperitoneal injection of PBS, PTX (20 μg/kg), or LPS (1 mg/kg). The means were compared using ANOVA (*P*-values as indicated) followed by *post hoc *Student's *t *tests. *Significantly different from the corresponding isotype group. *n *= 6-10 per group. ***c***, Data from an experiment similar to the one shown in *b*, except that LPS-challenged mice received a higher dose of anti-CXCL1 antibody (20 mg/kg instead of 4 mg/kg) or a combination of anti-CXCL1 and anti-CXCL2 antibodies (4 mg/kg each). *n *= 7 per group.

We have previously reported that the population of adherent intraluminal leukocytes (CD45^high ^cells with a round or rod-shape morphology) increases in the cerebral vasculature after exposure to bacterial toxins, and that this response is largely attributable to *de novo *recruitment of granulocytes [17]. To examine the importance of CXCL1 in this response, we pretreated mice with 4 mg/kg anti-CXCL1, anti-CXCR2 (positive control), or isotype antibody (negative control) before challenging them with PTX or LPS. Histological analysis revealed that the increase in rod-shaped leukocytes in the vasculature of the cerebral cortex was reduced by ~69% by the anti-CXCL1 and anti-CXCR2 antibodies in mice exposed to PTX, but was reduced to a similar extent only by the anti-CXCR2 antibody in mice exposed to LPS (Figure 4a, b). In contrast, the number of round leukocytes was not affected by any treatment. To verify whether the lack of effect of the anti-CXCL1 antibody in LPS-treated mice was due to a compensatory effect of CXCL2 or the use of a suboptimal dose of anti-CXCL1 antibody, we repeated the analysis with LPS-challenged mice pre-treated with either a combination of anti-CXCL1 and anti-CXCL2 antibodies (both at 4 mg/kg) or a higher dose of anti-CXCL1 antibody (20 mg/kg). We observed a reduction in the recruitment of rod-shape leukocytes only with the latter treatment (Figure 4c), suggesting that LPS induces a higher increase in CXCL1 than PTX and that a higher dose of anti-CXCL1 antibody is required for effective pharmacological blockade. This is supported by the observation that increased serum levels of CXCL1 were detected by ELISA in LPS-treated mice, but not in PTX-treated mice (LPS, 1.3 ng/ml; PTX, 0.40 ng/ml; PBS, 0.33 ng/ml; ANOVA, *P *= 0.019). Finally, to exclude a possible effect of the antibodies on the circulating pool of granulocytes, we analyzed blood samples collected at the time of sacrifice by flow cytometry. No significant difference in the number of CD45^+^7/4^+^Ly6G^+^CD115^- ^cells was found among the animals that received the different antibodies (data not shown; ANOVA, *P *= 0.5), suggesting that the anti-CXCL1 and anti-CXCR2 antibodies blocked the adhesion of granulocytes, but not their mobilization or survival.

**Figure 5:**
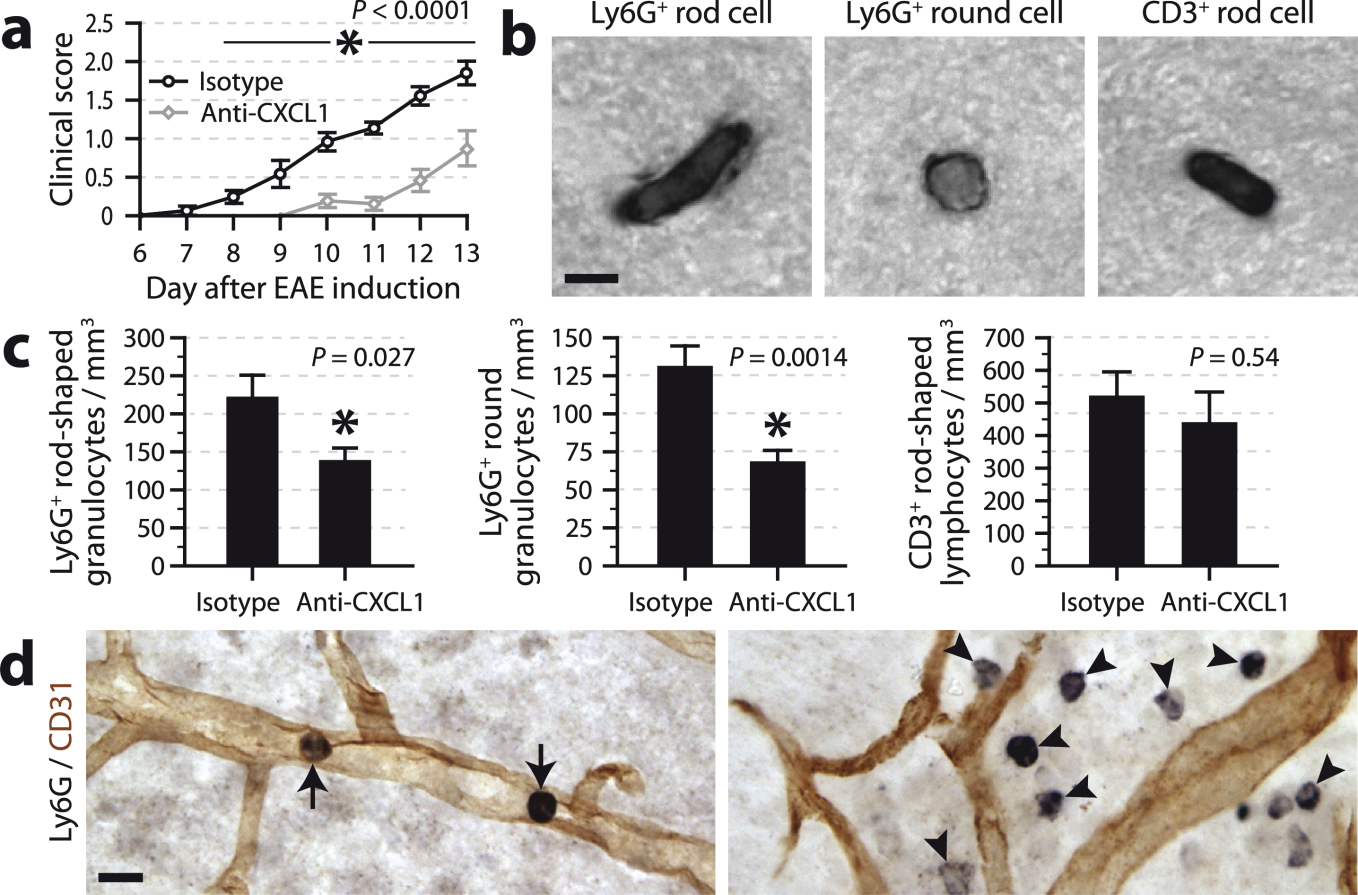
**Contribution of CXCL1 to the development of EAE**. ***a***, Clinical scores of EAE mice injected intravenously once daily from day 7 with 4 mg/kg anti-CXCL1 antibody or isotypic control antibody. The means were compared by multivariate ANOVA with repeated measures (*P*-value as indicated) followed by *post hoc *Student's *t *tests. *Significantly different from the isotype group. *n *= 9-11 per group. ***b***, Micrographs of different subsets of leukocytes stained for Ly6G or CD3 by immunohistochemistry in brain sections from mice killed 13 days after EAE induction. Scale bar = 5 μm. ***c***, Counts of leukocytes in the cerebral cortex of EAE mice treated or not with anti-CXCL1 antibody and killed at day 13. The means were compared using the Student's *t *test. *Significantly different from the isotype group. *n *= 9-11 per group. ***d***, Micrographs of granulocytes located inside (arrows) or outside (arrowheads) brain capillaries in a EAE mouse. Black = Ly6G immunoperoxidase staining using nickel-DAB as a substrate. Red-brown = CD31 immunoperoxidase staining using DAB as a substrate. Scale bar = 10 μm.

To assess whether CXCL1 contributes to EAE development by promoting granulocyte recruitment, we injected mice once daily with 4 mg/kg anti-CXCL1 antibody from day 7 to day 13 after EAE induction by active immunization. Blind evaluation of the clinical symptoms revealed a reduction in EAE severity in these mice compared to sham-treated animals (Figure 5a). All the mice were killed 4 h after the last injection to collect their brains for histological analyses. Because the populations of intravascular rod-shaped leukocytes found in the brains of EAE mice comprises not only granulocytes but also T lymphocytes [17], we distinguished these cells by using antibodies against the granulocyte marker Ly6G and the T cell marker CD3 (Figure 5b). Stereological cell counts showed a 38% reduction in the number of Ly6G^+ ^rod-shaped cells, but no significant change regarding the subpopulation of CD3^+ ^rod-shaped cells (Figure 5c) or the total population of CD3^+ ^cells (isotype antibody, 875 ± 183/mm^3^; anti-CXCL1 antibody, 971 ± 193/mm^3^). Also reduced was the population of Ly6G^+ ^rounded granulocytes (Figure 5c), which was > 4 times greater than that found in mice treated with bacterial toxins and included not only intraluminal, but also intraparenchymal cells (Figure 5d). Overall, this last series of experiments demonstrates that CXCL1 plays an important role in different neuroinflammatory conditions by promoting granulocyte adhesion to the cerebral microvasculature.

## Discussion

As growing evidence suggests that granulocytes contribute to EAE and perhaps multiple sclerosis, there is high interest in understanding how these cells are recruited in the CNS. While the cytokines and adhesion molecules involved in this recruitment have started to be identified (e.g., IL-6, IL-1β, TNF, ICAM1, Mac1) [17,21], the required chemokine was undetermined prior to the present study. Our results reveal that CXCL1 is a major CXCR2 ligand upregulated in cerebral endothelial cells at least by IL-6 in different inflammatory conditions. They also show that CXCL1 plays an essential, non-redundant role in the recruitment of granulocytes by promoting their adhesion to capillaries. Finally, and as discussed below, our results not only help to understand the mechanism of action of PTX and the importance of granulocytes in EAE, but provide a pre-clinical validation for the use of CXCL1 inhibitors for the treatment of neuroinflammatory disorders.

### Mechanism of action of PTX

**Figure 6:**
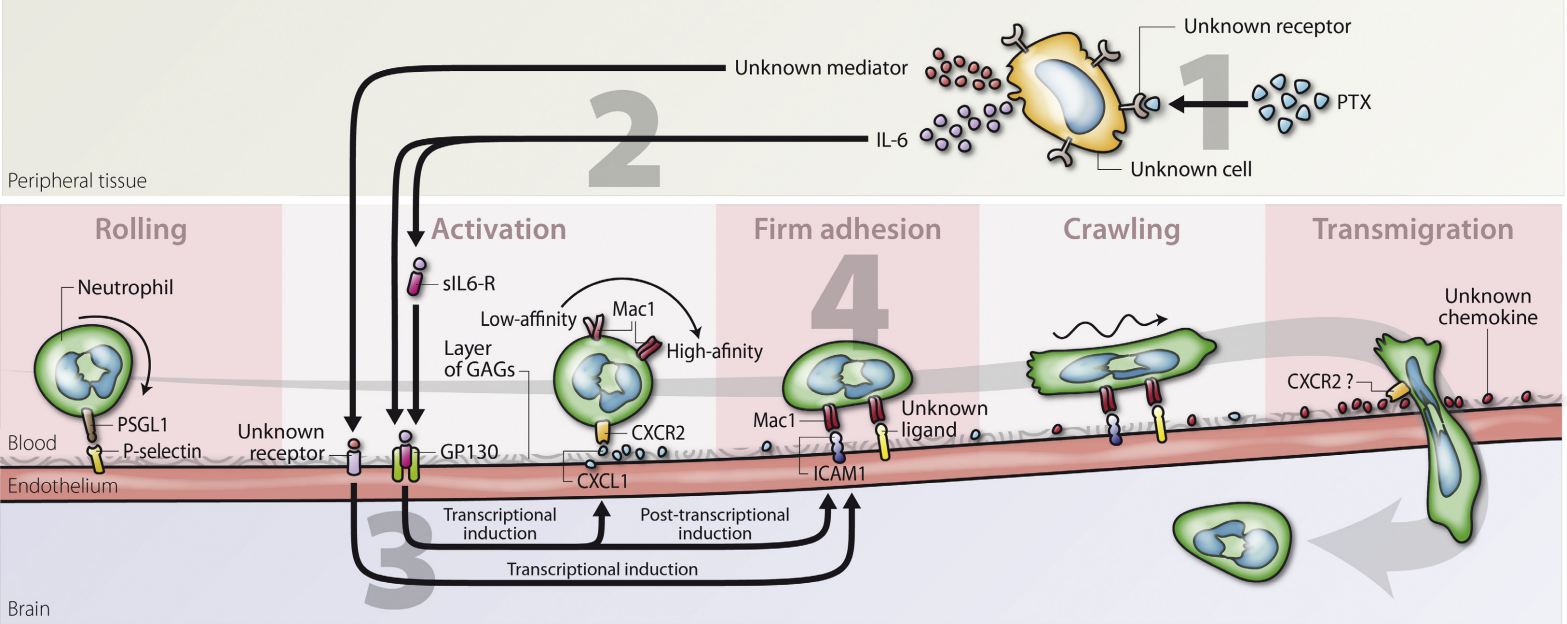
**Mechanism of granulocyte recruitment at the blood-brain interface during PTX exposure**. The present results, together with those from a previous study [17], allow us to propose the following mechanism: 1) PTX induces IL-6 expression in peripheral cells through a receptor that remains to be identified; 2) IL-6 travels in the blood and stimulates CXCL1 expression in endothelial cells by acting transcriptionally; 3) IL-6 also stimulates ICAM1 expression by acting post-transcriptionally and in synergy with a second mediator acting transcriptionally; 4) granulocytes are activated by the binding of CXCL1 to CXCR2 and adhere to the endothelium through interaction between Mac1 and ICAM1.

To induce EAE, the most studied animal model of multiple sclerosis, mice immunized with myelin antigens or transplanted with myelin-reactive T cells are commonly injected with PTX, a multimeric protein produced by the bacteria causing whooping cough and used as an adjuvant to increase EAE incidence and severity [31,41]. The mechanism by which PTX promotes EAE is still not fully understood and seems paradoxical considering its ability to block leukocyte migration by interfering with G protein-coupled receptor signaling [42]. We have recently published evidence that PTX increases endothelial adhesiveness indirectly through circulating mediators such as IL-6 [17]. The latter induces ICAM1 expression in brain capillaries by acting post-transcriptionally, leading to the recruitment of leukocytes capable of patrolling the cerebrospinal vasculature by crawling on its luminal surface. As illustrated in Figure 6, the present study extends this concept by demonstrating that IL-6 is also responsible for the upregulation of a chemokine that promotes granulocyte adhesion and EAE development, this time by acting transcriptionally. To complete our model, it will now be interesting to identify the chemoattractant that informs granulocytes on where to penetrate the CNS parenchyma, as well as to delineate the mechanism sensing PTX and initiating this entire inflammatory cascade.

### Importance of granulocytes and the CXCL1-CXCR2 axis in EAE

Although a beneficial role of CXCL1 and granulocytes may be drawn from a study showing that forced overexpression of CXCL1 in astrocytes decreases the severity of EAE [43], many studies in which CXCR2 was neutralized or granulocytes were depleted actually suggest the opposite [12,18-20]. In agreement with the latter studies, we show here that the number of adherent granulocytes and the severity of EAE were reduced in mice treated with an anti-CXCL1 antibody during the effector phase of the disease, suggesting that CXCL1 significantly contributes to EAE by promoting granulocyte recruitment. As recently reviewed [44], granulocytes may play different roles in EAE, such as the secretion of immunoregulatory molecules that would sustain the activity/recruitment of other immune cells and the execution of effector functions that would damage the myelin sheath. It will be important in future work to test these possibilities, but this will require the development of new granulocyte-specific genetic mouse models.

### CXCL1 as a potential therapeutic target

Genetic deletion or pharmacological blockade of CXCR2 has been shown to inhibit EAE [12,19,20]. Considering that CXCR2 can bind to several ligands, one can predict that blocking this receptor would be less specific and more immunocompromising than blocking CXCL1. It may thus be advantageous to develop CXCL1 inhibitors that would allow the action of the other CXCR2 ligands, which might be significantly produced under various conditions. The present study supports the latter approach by showing that an anti-CXCL1 antibody can improve the clinical symptoms of EAE, although the improvement we observed was lower than that obtained in other laboratories by targeting CXCR2 [12,19]. Different reasons might explain the lower effectiveness of our anti-CXCL1 treatment, such as the use of a non-optimal dose or frequency of injection, and a loss of activity over time due to the establishment of an anti-drug immune response. Developmental research should be considered to optimize the therapeutic use of anti-CXCL1 antibodies and to determine whether other anti-CXCL1 inhibitors could be advantageous. Furthermore, the importance of examining whether blocking CXCL1 would be safe in the long term and efficient in all types of EAE and multiple sclerosis is underscored by the observations that CXCL1 influences the biology of myelinating cells [45-48] and that both CXCL1 expression and neutrophil infiltration are higher during Th17 cell-mediated EAE compared to Th1 cell-mediated EAE [12,13].

## Conclusions

This study highlights the importance of the CXCL1-CXCR2 axis in the recruitment of granulocytes at the blood-brain interface and in the development of EAE. The challenges are now to determine whether other CXCR2 ligands exert a significant influence on these processes despite lower expression levels, to elucidate the precise functions of granulocytes in demyelinating diseases, and to translate this knowledge into new therapeutic strategies for multiple sclerosis.

## Abbreviations

(CNS): Central nervous system; (EAE): experimental autoimmune encephalomyelitis; (ICAM1): intercellular adhesion molecule 1; (IL-6): interleukin-6; (IL-6Rα): IL-6 receptor α; (LPS): lipopolysaccharide; (PBS): phosphate-buffered saline; (PTX): pertussis toxin; (TNF): tumor necrosis factor.

## Competing interests

The authors declare that they have no competing interests.

## Authors' contributions

LV designed the experiments, supervised the project, and wrote the paper. MR and JFR performed most of the experiments with the assistance of AD. All authors read and approved the final manuscript.

## Supplementary Material

Additional file 1 **Figure S1**. Mean clinical scores of EAE mice used in the experiment shown in Figure 1 on the day of sacrifice. *n = *6 (days 3, 6, and 10), 5 (day 8), or 3 (day 12). Click here for file
