# Supplementary figures and images for: CXCL1 can be regulated by IL-6 and promotes granulocyte adhesion to brain capillaries during bacterial toxin exposure and encephalomyelitis
Source: J Neuroinflammation. 2012 Jan 23;9:18. doi: 10.1186/1742-2094-9-18 (PMC3283467; doi:10.1186/1742-2094-9-18)

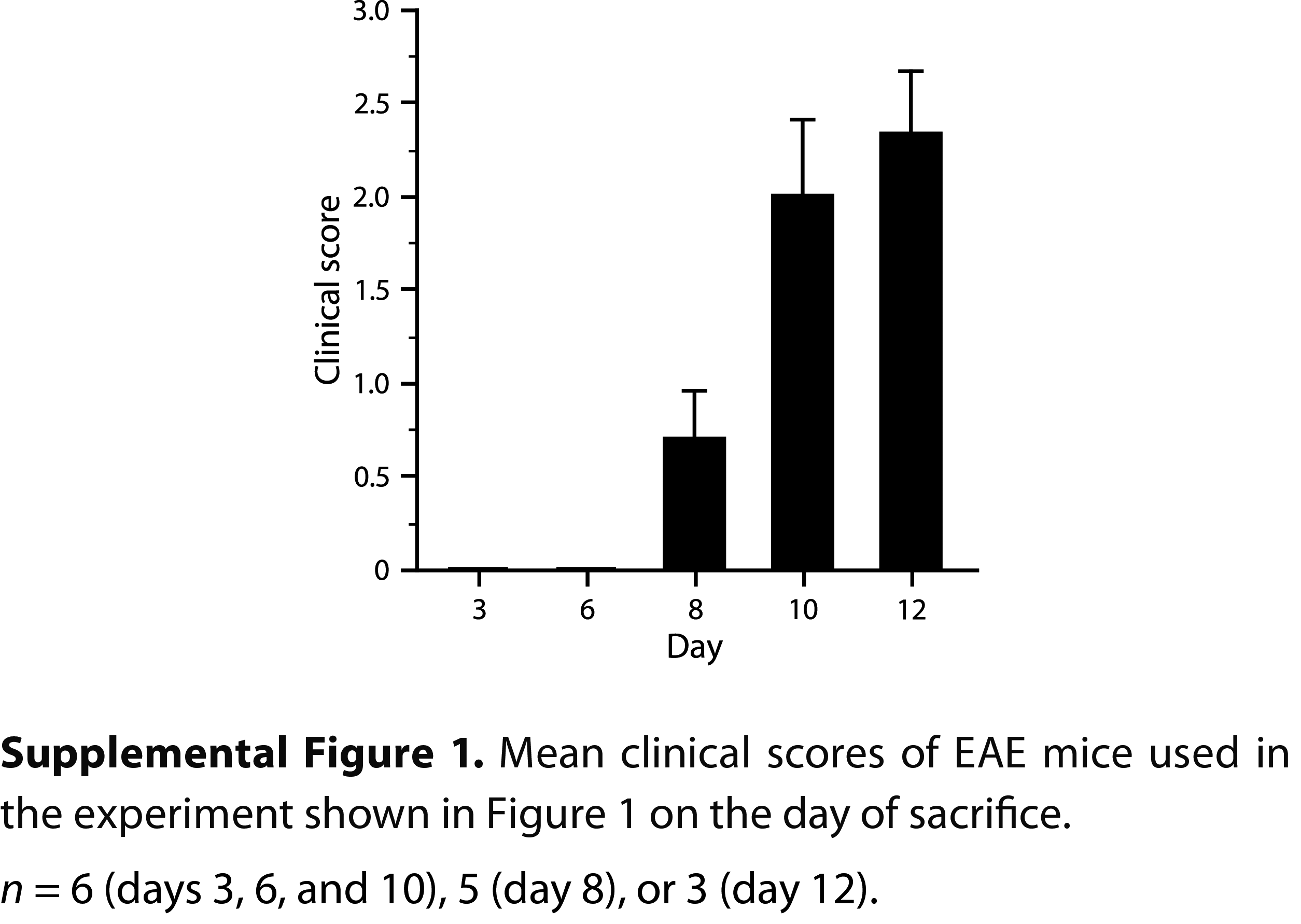

Supplement: Additional file 1 — Figure S1. Mean clinical scores of EAE mice used in the experiment shown in Figure 1 on the day of sacrifice. n = 6 (days 3, 6, and 10), 5 (day 8), or 3 (day 12). [file 1742-2094-9-18-S1.TIFF]
